# Book Reviews

**Published:** 1898-08

**Authors:** 


### Book Reviews.

Twentieth Century Practice. An International Encyclopedia of Modern Medical Science. By leading authorities of Europe and America. Edited by Thomas L. Stedman, M. D., New York City. In twenty volumes. Vol. XIV., Infectious Diseases. New York: William Wood & Co. 1898.

This volume treats of the infectious diseases and opens with scarlet fever, by Forchheimer, who always writes interestingly, but never more so than on this occasion. Measles is next taken up by Dawson Williams, who has given us the latest information concerning this common disease of childhood and he is especially strong concerning its complications. Short chapters, then, follow on German measles, by Forchheimer, chickenpox, by Dillon Brown, and glandular fever, by Dawson Williams. The next chapter deals with whooping cough and is written by Joseph O'Dwyer and Nathaniel Read Norton. This disease is not as well understood as to etiology and treatment as it should be, but whatever is known is here recorded. The next subject is cholera infantum, which is handled by Jacobi in his usual masterly manner. This chapter is especially important at this season of the year. Cholera nostras is the next subject and is dealt with by Theodor Rumpf, and he, also, takes up Asiatic cholera in the succeeding chapter, which is exhaustively presented. Dengue is the next disease considered and this chapter is written by Sir Joseph

Fayrer. It is an interesting exposition of a disease that is little understood. We next find beriberi taken up by Sodre, a disease upon which he throws much additional light. Military fever, by Netter, forms the subject of the next chapter and Malta fever, by Bruce, concludes the volume. A well-arranged index is added.

The book is uniform in style and size with its predecessors and, as will be seen by the foregoing synopsis, furnishes an interesting link in the chain contemplated by this series.

---

YELLOW FEVER. Clinical Notes. By JUST TOUATRE, M. D. (Paris); Member Board of Experts Louisiana State Board of Health. Translated from the French by Charles Chassaignac, M. D., President New Orleans Polyclinic; Editor New Orleans Medical and Surgical Journal. New Orleans: New Orleans Medical and Surgical Journal, Limited. 1898.

The subject of yellow fever has become of greater interest since our war with Spain began and especially since the disease has invaded our army in Cuba. Spain has been guilty for ages through direction and indirection of propagating this infection, and the world at large will owe the United States a debt of gratitude when it shall have banished from the western hemisphere, a misrule that for more than three centuries has been a mockery to civilisation and a shock to science.

The author and the translator of this treatise both have had exceptional opportunities for the clinical study of this disease. Touatre wrote the book in French because he distrusted his knowledge of English ; but it has been faithfully translated by Chassaignac, whose thorough knowledge of French idiom peculiarly fitted him for the task, in the exercise of which he has reproduced with exactitude the author's ideas.

Since the infection of yellow fever has been better understood the type of the disease has grown milder, the deaths fewer and the recoveries prompter. A vast amount of information bearing upon all phases of the malady is condensed into this little volume, the study of which we commend to all persons interested in the subject.

---

ORTHOPEDIC SURGERY. By JAMES E. MOORE, M. D., Professor of Orthopedia and of Clinical Surgery in the College of Medicine of the University of Minnesota; Fellow of the American Surgical Association; Member of the American Orthopedic Association; Surgeon to St. Barnabas Hospital, Minneapolis, etc. Octavo volume of 360 pages, illustrated with 177 half-tones, from photographs made specially for this work. Philadelphia: W. B. Saunders, 925 Walnut street. 1898.

The importance of a knowledge of the branch of surgery dealt with in this book is second to none. Indeed, the surgeon who seizes upon a little sufferer, corrects his deformity, restores him to health and makes him self-supporting, performs an act that entitles him to the gratitude of mankind everywhere. There is no more philanthropic work that can engage the attention of the surgeon than the correction of deformities, whether of childhood or adult age.

This author has presented the subject in concise form and has adapted his treatise especially to the use of students and general practitioners of medicine. The illustrations are, for the most part, original and serve to elucidate a clear understanding of the text. Dr. Moore is a general surgeon, but has taught orthopedic surgery for the past ten years, hence his statements have the weight of experience. The treatise is commended to all interested in the subject. Its mechanical execution is unsurpassed.

---

Transactions of the American Association of Obstetricians and Gynecologists. Volume X. For the year 1897. Edited by William Warren Potter, M. D., Secretary. Octavo, pp. viii.—483. Philadelphia: William J. Dornan. 1898.

This handsome volume contains the proceedings of this association at its tenth annual meeting, held at Niagara Falls, August 17-20, 1897. Its contents abound in interesting papers and admirable discussions. The association appears to outdo itself year by year in the excellence of its work and the method of its presentation; at all events, though many excellent volumes have appeared, none are better than this one. Among other things it contains well-prepared memoirs of Mr. James Greig Smith, Dr. Traill Green and Dr. Fletcher D. Mooney, each of which is accompanied by a handsome life-like portrait.

The next meeting of the association is announced for Pittsburg, September 20-22, 1898, under the presidency of Dr. Charles A. L. Reed, of Cincinnati.

---

The Treatment of Choleraic Diarrhea. Published by the Lambert Pharmacal Company, St. Louis. 1898.

This useful little brochure of sixty-four pages is a compilation of experiences in the treatment of summer diarrhea in childhood and adult life by prominent medical men throughout the country. It is seasonable in its appearance as we are just now in the weather belt of enteric disease, and its contents should prove useful to everyone called upon to treat these affections.

---

Hay Fever and Its Successful Management. By W. C. Hollopeter, A. M., M. D., Clinical Professor of Pediatrics in the Medico-Chirurgical College of Philadelphia; Physician to the Methodist Episcopal Hospital, etc., etc. Duodecimo, pp. 137. Philadelphia: P. Blakiston, Son & Co. 1898.

The season is near upon us when the sufferers from hay fever will be seeking relief, hence it is opportune that this little treatise should appear at this time. It is well written and discusses the subject from the viewpoint of science. It gives the history of the disease, its exciting and predisposing causes, the time of its occurrence and its duration, its symptoms, complications and sequelæ, its pathology, diagnosis, prognosis and treatment—all in consecutive order. It contains a most complete bibliographical list, arranged in chronological order from 1565 to 1898. A well-arranged index closes one of the best essays on the subject that we have lately seen and we hope the author's implied promise of successful treatment will be realised for the sake of both physician and patient.

